# Prognostic Significance of RAS Mutations and P53 Expression in Cutaneous Squamous Cell Carcinomas

**DOI:** 10.3390/genes11070751

**Published:** 2020-07-06

**Authors:** Manuel António Campos, Sofia Macedo, Margarida Sá Fernandes, Ana Pestana, Joana Pardal, Rui Batista, João Vinagre, Agostinho Sanches, Armando Baptista, José Manuel Lopes, Paula Soares

**Affiliations:** 1 Instituto de Investigação e Inovação em Saúde, Universidade do Porto, 4200-135 Porto, Portugal; manuelantonioccampos@gmail.com (M.A.C.); sofiamacedo_92@hotmail.com (S.M.); apestana@ipatimup.pt (A.P.); rbatista@ipatimup.pt (R.B.); jvinagre@ipatimup.pt (J.V.); jmlopes@ipatimup.pt (J.M.L.); 2 Institute of Molecular Pathology and Immunology of the University of Porto (Ipatimup), 4200-135 Porto, Portugal; 3 Medical Faculty, University of Porto, 4200-450 Porto, Portugal; 4 Department of Dermatology, Centro Hospitalar Vila Nova de Gaia, 4434-502 Vila Nova de Gaia, Portugal; abaptista@chvng.min-saude.pt; 5 Department of Pathology, Centro Hospitalar São João, 4200-450 Porto, Portugal; margarida.mi@gmail.com (M.S.F.); joana_pardal@hotmail.com (J.P.); 6 Department of Pathology, Centro Hospitalar Vila Nova de Gaia, 4434-502 Vila Nova de Gaia, Portugal; sanchesagostinho@gmail.com

**Keywords:** *RAS*, p53, mutation, expression, prognostic biomarker, prognosis, biomarker, cutaneous squamous cell carcinoma, recurrence, metastases, outcome

## Abstract

*TP53* is considered the most commonly-altered gene in cutaneous squamous cell carcinoma (cSCC). Conversely, *RAS* mutations have been reported in a low percentage of cSCC. The objective of our study was to evaluate the frequency of p53 expression and *RAS* mutations in cSCC and correlate them with clinicopathological features and patient outcome. We performed immunohistochemistry for p53 and genetic profiling for *RAS* mutations in a retrospective series of cSCC. The predictive value of p53 expression, *RAS* mutations, and clinicopathological parameters was assessed using logistic regression models. The overall frequency of *RAS* mutations was 9.3% (15/162), and 82.1% of the cases (133/162) had p53 overexpression. *RAS* mutations rate was 3.2% (1/31) of in situ cSCCs and 10.7% (14/131) of invasive cSCCs. *RAS* mutations were more frequently associated with an infiltrative than an expansive pattern of invasion (*p* = 0.046). p53 overexpression was a predictor of recurrence in the univariate analysis. Our results indicate that *RAS* mutations associate with features of local aggressiveness. Larger studies with more recurrent and metastatic cSCCs are necessary to further address the prognostic significance of p53 overexpression in patients’ risk stratification.

## 1. Introduction

Cutaneous squamous cell carcinoma (cSCC) is the second-most-common skin cancer in Caucasians and cumulative ultraviolet radiation is considered the major ethiopathogenic factor [1,2]. cSCC carcinogenesis includes premalignant lesions (actinic keratosis (AK) and in situ squamous carcinoma/Bowen’s disease), invasive, and metastatic cSCCs, although a multistep model is not always detected [3]. Some studies state that 65% of cSCCs arise from AK [3]. cSCCs most frequently occur in chronically sun-exposed areas such as the face (particularly the lip, ear, nose, cheek, and eyelid) and the dorsum of the hands. In order to assist prognostics, cSCCs are classified based on their histological subtype (e.g., acantholytic, spindle, verrucous, and desmoplastic), grade of differentiation (well differentiated, moderately differentiated, poorly differentiated, or undifferentiated), tumor depth (maximum vertical thickness), level of dermal invasion (Clark’s level) and the presence/absence of perineural, lymphatic, or vascular invasion [4]. Even though not optimal for cSCCs, to date staging is based on the TNM (Tumor, Node, Metastasis) system of the 2010 American Joint Committee on Cancer (AJCC) guidelines [5] and for head and neck cutaneous squamous cell carcinoma the recent 8th edition of the AJCC [6]. cSCCs can recur (3–5%) and metastasize (4–5%) [7]. Patients with localized cSCCs usually have an excellent outcome but for metastatic cSCCs a poorer prognosis is observed with survival rates of 25–35% (five-year survival rate) and less than 10% (ten-year survival rate) [8,9,10]. Only clinicopathological prognostic markers have been reported in cSCC for recurrence (tumor thickness > 2 mm and >6 mm, invasion beyond subcutaneous fat, perineural invasion, tumor size > 2 cm, and poor differentiation and location in the temple) and metastasis (tumor thickness > 2 mm and >6 mm, invasion beyond subcutaneous fat, perineural invasion, tumor size > 2 cm, poor differentiation, immunosuppression, and location in the temple, lip, and ear) [11]. We recently reported the association of *TERT* promoter mutations with worse prognosis (recurrence and metastasis) but we admit that its putative prognostic significance still needs to be established in larger series [12].

The *TP53* gene encodes a nuclear transcription factor that is usually involved in the negative regulation of the cell cycle and in promoting apoptosis and is frequently impaired during tumor progression [13,14,15]; it has been considered the most-commonly-mutated gene in squamous cell carcinoma. Immunohistochemical expression of p53 has for a long time been a matter of debate in cSCC [16]. p53 overexpression varies greatly among different studies (15–92%) [17] and was associated with either wild type or mutated cases [18,19]. It is suggested that the immunopositivity of p53 is not a surrogate marker of *TP53* mutation [18,19] and that p53 overexpression seems to be precocious in chronic sun-exposed skin, sometimes preceding genomic instability [17,20]. Several mechanisms were suggested to regulate p53 expression. MDM2 has been hypothesized as a strong modulator of p53 ubiquitination and its modulation could result in increased p53 expression [21,22]. Another potential mechanism for p53 overexpression is aberrant p53 protein accumulation due to tetrameric proteins formed by wild type and mutant p53 proteins, the well-known dominant negative effect [23]. The prognostic impact of p53 overexpression in cSCC demands further clarification.

*RAS* is a small GTPase that activates the mitogen-activated protein kinases (MAPKs) and other signaling pathways involved in cell survival, proliferation, and apoptosis. *RAS* mutations were reported in a low percentage of cSCCs (<13%) [24,25,26,27]. A significant subset of patients treated with either the multikinase inhibitor sorafenib or the *BRAF* V600E inhibitors, vemurafenib and dabrafenib, rapidly develop cSCCs harboring *H-RAS* mutations [28]. This evidence points to the fact that these inhibitors give rise to paradoxical activation of the MAPK pathway, which in turn cooperates with mutations in other key oncogenes and tumor suppressors such as *H-RAS* and *TP53* [29]. These recent data have drawn attention to the role of *RAS* mutations in cSCC carcinogenesis.

In vivo studies revealed that a germline *TP53* mutation and activated *H-RAS* act synergistically to enhance tumor progression [30]. Taking into account the possible interplay between *TP53* and *RAS*, we assessed p53 overexpression, *H-RAS,* and *K-RAS* mutations in a large series of cSCCs and correlated these alterations with clinicopathological features and patients´ outcome.

## 2. Materials and Methods

### 2.1. Patient Selection, Sample Selection, and Clinicopathological Characterization

All the procedures reported in this study were in accordance with national and institutional ethical standards and were approved by the Local Ethical Review Committees of the Centro Hospitalar Vila Nova de Gaia e Espinho (CHVNGE) (ethical permit number 182-2014-3 with the title “Carcinogénese do carcinoma espinocelular da pele” attributed to M.A.C.). According to Portuguese law, informed consent is not required for retrospective studies.

The descriptive and statistical analysis refers to all the consecutive cSCCs surgically removed at CHVNGE within the time period between January 2004 and December 2013. For the inclusion criteria, we selected immunocompetent patients with a histological diagnosis of cSCC and with available follow-up data. Exclusion criteria were applied to patients with genetic diseases that conferred increased risk of cSCC, such as xeroderma pigmentosum, epidermodysplasia verruciformis, and albinism. None of the cases of this retrospective series was subjected to Mohs micrographic surgery. Cases with available formalin-fixed paraffin-embedded tissues (FFPE) were retrieved from the Pathology Department of CHVNGE. One hundred and eighty-four histological specimens were reclassified by pathologists experienced in cutaneous neoplasms (J.M.L., J.P., and M.F.). Tumors were evaluated based on the protocols from the College of American Pathologists (CAP) and the American Joint Committee on Cancer (AJCC) guidelines [31,32]. The evaluated CAP criteria were tumor site and size, histological type and grade, thickness, status of surgical (superficial and deep) margins of the excised cSCC, lymph-vascular and perineural invasion, lymph node status, and pathological staging (pTNM). We included further criteria, such as the pattern of infiltration (expansive or infiltrative), presence of ulceration, peritumoral and intratumoral lymphocytic infiltrate, and the presence of AK in adjacent skin. Transected tumor biopsies of 32 cases (4 in situ cSCCs and 28 invasive cSCCs) were genetically and immunohistochemically profiled to evaluate primary tumors without alterations (e.g., fibrosis) derived from subsequent complete re-excision of previously-biopsied tumors. No evidence of invasive components was detected in any of the re-excised specimens of the in situ cSCCs. Similarly, the values of the parameters evaluated in the invasive cSCC cases did not differ (data not shown). None of the 32 cases was used to evaluate the impact of surgical margins, except for the other parameters (as stated in the Results and Tables), whenever they were adequately assessed. Representative areas were selected by the pathologists in hematoxylin and eosin slides to continue with microdissection.

Age at diagnosis was registered and stratified into two groups according to the mean age (<80 years vs. ≥80 years) for statistical analyses. Topographic locations of the tumors were classified according to the International Statistical Classification of Diseases and Related Health Problems 10th Revision (including lips, eyelid, ear, face, scalp/neck, trunk, upper limb, lower limb, and not specified) [33]. The topographic locations were stratified into five locations, namely, the face (including the neck), trunk, upper limb, lower limb, and not specified. Topographic locations were additionally subdivided into chronically sun-exposed (scalp/neck, face, ears, eyelids, and hands) and intermittently sun-exposed (trunk, upper limb, lower limbs, and feet) locations. The tumors were divided into in situ and invasive cSCCs; the latter were subdivided by histologic type (acantholytic, spindle cell, verrucous, pseudo vascular, adenosquamous, and not otherwise specified (NOS)). The histological grade was classified in well-, moderate-, or poorly-differentiated and undifferentiated. Pattern of invasion was divided in expansive or infiltrative. The tissue level of the tumor invasion was classified as invading the papillary dermis, the reticular dermis, the subcutaneous tissue, or beyond. The maximum tumor thickness of invasive cSCCs was split into two groups <6 and ≥6 mm; distance to the nearest superficial and deep surgical margins were also measured in mm. The presence of ulceration and actinic keratosis was annotated; actinic keratosis was evaluated in the adjacent skin. Intra- and peritumoral lymphocytic infiltrate was classified as moderate–intense or few–absent. The presence of lymphovascular and perineural invasion was annotated. “T” of each tumor was classified according to the TNM classification. Recurrence was defined as the development of a histologically-confirmed cSCCs in the same topographic area in addition to being identified by the assisting dermatologist as recurrence, as in previous studies [7,34]. Progression-free survival (PFS) was defined as the time until diagnosis of recurrence and/or metastasis; PFS and overall follow-up are presented in months.

### 2.2. DNA Extraction and Mutation Analysis

DNA was extracted from 10-µm cuts of FFPE tissues followed by microdissection. The DNA extraction kit (Citogene^®^, Citomed, Portugal) was used in accordance with manufacturer’s protocol. PCRs were performed with PromegaGoTaq^®^ G2 Flexi DNA polymerase (Promega, Southampton, UK) and with the recommended settings. Sanger sequencing was achieved using the BigDye Terminator Kit (Perkin-Elmer, CA, USA) and with the fragments running in an ABI (Applied Biosystems) prism 3100 Genetic Analyzer (Perkin-Elmer, CA, USA). Independent PCR amplification and sequencing were performed for both positive and inconclusive (not confirmed as positive or negative) samples. Sequencing analysis targeted exons 1 and 2 of *H-RAS* and *K-RAS*. The sequencing reactions were performed in a sense direction for exon 1 and in anti-sense direction for exon 2. Mutations were evaluated and classified using the Mutation Surveyor DNA variant analysis software (Softgenetics, PA, USA) and were matched to reference control sequences from GenBank.

### 2.3. Immunohistochemistry Protocol and Analysis

Sections, 4-µm in thickness, from the FFPE blocks, were used for immunohistochemical studies. Specimen tissues were deparaffinized and rehydrated. The antigen retrieval was performed on a steamer for 45 min using citrate buffer pH 6.0 from Thermo Scientific (TA-050-CBX). Endogenous peroxidase was blocked, and a non-specific binding blockage from Thermo Scientific (TA-125-PQB) was also used. Sections were incubated with the primary antibody, anti-p53 antibody (1:700) from Leica (NCL-L-p53-DO7) using a diluent from Thermo Scientific (TA-125-ADQ), during 60 min at room temperature. Then, a biotinylated goat polyvalent secondary antibody was used from Thermo Scientific (TP-125-BN). Finally, the chromogenic detection was performed with 3,3′-diaminobenzidine (Dako, K3468) reaction and counterstained with Mayer’s hematoxylin. Negative controls underwent a similar procedure, with the exclusion of the primary antibody.

**Figure 1:**
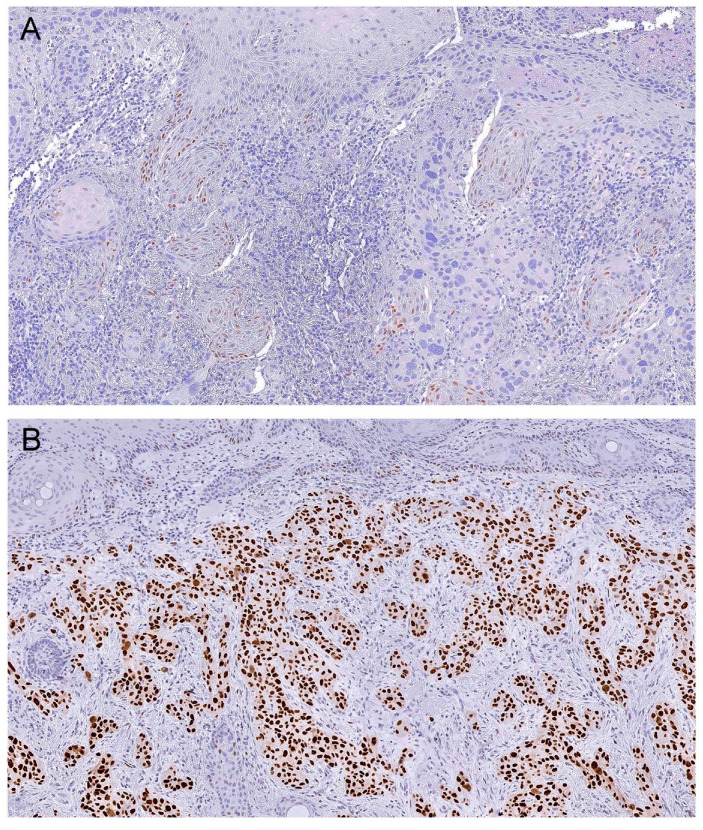
Representative images of immunoexpression of p53 in cutaneous squamous cell carcinomas (cSCCs) for (**A**) wild type expression and (**B**) overexpression. Original magnification 100×.

Each slide underwent digital acquisition using a D-Sight Fluo 2.0 digital microscope (A. Menarini Diagnostics, Florence, Italy). Tumor area to be analyzed was manually selected on the digital slide by one researcher (M.A.C.) until at least 2000 tumor cells were included. Whenever the whole tumor specimen could not be selected, the invasive front of the tumor was preferentially included. The same digital slide was scanned with a validated automated scanning system (VISIA Imaging s.r.l. software version 2.5.0.1, Italy) [35]. The software automatically established the overall percentage of positive cells and the percentage of cells that disclosed absent staining (0), weak staining (1+), moderate staining (2+), and strong staining (+3). The software additionally calculated an “h-index score” (sum of the percentage of positive cells * staining intensity). The h-index score (h-score) is a continuous variable that represents the mean score of positivity and intensity. p53 expression was considered as normal (wild type) when few/scattered dispersed cells disclosed immunostaining, and abnormal when overexpression was present (see Figure 1). p53 overexpression was quantified using an h-score that had been previously reported as a valid approach for routine immunohistochemical quantification [36,37]. H-score was defined by the software as 1+ (h-score < 100), 2+ (h-score 100–200), and 3+ (h-score 200–300). The expression in non-tumor cells (e.g., epidermis, follicular, and adnexal glandular epithelium, and mesenchymal cells) present in each case was used as an internal control for wild type p53 expression. A certified dermatopathologist (J.M.L.) performed an internal validation of our series without prior consultation or recourse to clinical, or previously-annotated p53 h-score data, as reported in other studies [38], and confirmed an agreement between this new method and routine standard immunohistochemistry analysis (data not shown).

### 2.4. Statistical Analysis

Continuous variables were described by the mean and standard deviation (SD); the categorical variables were described by the absolute frequency and the relative frequency. Differences in proportions were tested with the chi-square test or the Fisher’s exact test, as appropriate. Differences between two independent samples were assessed with the t test for continuous variables. Analysis of variance (ANOVA) was used to compare continuous variables between independent samples. The predictive value of *RAS* mutation, p53 expression, and other parameters for recurrence, metastasis, and PFS was assessed using univariate and multivariate logistic regression models. In the logistic regression models, all the parameters that were significantly associated with the outcome in the univariate model were included in the multivariate analysis. The odds ratio (OR) and respective 95% confidence intervals (CI) were estimated in the regression models. The results were considered statistically significant at *p* < 0.05. The statistical analysis was conducted with software Statistical Package for the Social Sciences (SPSS) version 24.0 (SPSS Inc., Chicago, IL, USA).

## 3. Results

Of the 184 histologically-characterized cases, *RAS* and p53 status was not determined in 22 cases due to the small size and/or low quality of the tumor samples. For the remaining 162 lesions, excised from 128 patients, we analyzed *RAS* mutations and p53 expression, Table 1. Of these, 31 were in situ cSCCs and 131 invasive cSCCs, Table 1. The overall frequency of *RAS* mutations was 9.3% (15 out 162 cases) and the mean p53 overexpression was 1+ h-score (91.6 ± 5.9), Table 1. p53 overexpression was observed in 82.1% of the cases (74.2% of the in situ and 84.0% of the invasive cSCCs), Table 1. None of the cases had null-type expression, Table 1. The mean p53 h-score of in situ cSCCs was 109.6 ± 16.3 and 87.9 ± 6.2 in invasive cSCCs. 146 out of 162 cases (90.1%) had available *TERTp* mutation status previously published by our group and were included when appropriate [12], Table 1.

### 3.1. Relationship between RAS Mutations, P53 Expression, and Clinicopathological Features

Table 1 presents the clinicopathological features, the frequency of *RAS* mutations, and p53 expression in the series. Clinicopathological factors and their association with *RAS* mutations and p53 expression are presented in Supplementary Tables S1–S3.

We analyzed all cSCCs and observed that *RAS* mutations were present in lesions on the face, trunk, and upper and lower limbs. *RAS* mutations were more frequent in male than in female patients but without statistical significance. The *RAS* mutations did not associate with any clinicopathological feature of in situ cSCCs.

p53 overexpression was higher in women and on locations intermittently sun-exposed, but without statistical significance. p53 h-score was significantly higher in in situ cSCCs located on the lower limbs compared to other locations (161.8 ± 78.7 vs. 69.4 ± 49.9, respectively; *p* = 0.002). The three in situ cSCCs that recurred were wild type for *RAS* mutations and had p53 overexpression.

The *RAS* mutations were more frequently associated with an infiltrative than an expansive pattern (10 out of 57 (17.5%) vs. 4 out of 70 (5.7%), respectively; *p* = 0.046) in invasive cSCCs. Despite not reaching statistical significance, tumors with *RAS* mutations had more lymphovascular invasion than tumors with wild type *RAS* (2 out of 5 (40.0%) vs. 12 out of 126 (9.5%), respectively; *p* = 0.088). The *RAS* mutations were not associated with recurrence and metastasis of cSCC. p53 h-score was higher in recurrent than in non-recurrent tumors (118.8 ± 78.0 vs. 82.6 ± 61.5, respectively; *p* = 0.039).

*RAS* mutations and p53 overexpression were not associated with *TERTp* mutations in this series of cSCCs (see Supplementary Tables S1–S3).

### 3.2. Relationship between RAS Mutation, P53 Expression, and Outcome

For this analysis, we only included invasive cSCCs (*n* = 131). The mean follow-up time (± SD) of the patients was 42.2 ± 30.3 months (range 6–156 months).

Seventeen cases (13.0%) and 8 cases (6.1%) presented recurrence and/or metastasis, respectively (all were lymph node metastasis), during follow-up of patients. Supplementary Table S4 presents the main features of the cases with adverse outcomes.

A regression model was performed for parameters associated with an adverse outcome (recurrence or metastases) in invasive cSCCs (Table 2). When analyzing the parameters associated with the risk of recurrence, age > 80 years (OR 16.00; *p* = 0.008), presence of ulceration (OR 2.92; *p* = 0.049), and p53 overexpression (OR 1.01; *p* = 0.045) were identified as predictors in the univariate analysis. When the aforementioned parameters were included in the multivariate analysis, only age > 80 years (OR 12.17; *p* = 0.019) was identified as an independent predictor of recurrence.

When analyzing predictors of metastasis, the univariate analysis revealed that invasion of the subcutaneous tissue (OR 5.82; *p* = 0.028), distance to the nearest superficial margin (OR 1.18; *p* = 0.026), maximum tumor thickness (OR 1.25; *p* = 0.011), and few or absent peritumoral lymphocytes (OR 10.22; *p* = 0.032) were associated with a higher likelihood of metastasis. In the multivariate analysis, none of the aforementioned parameters was associated with a higher risk of metastasis of cSCCs.

## 4. Discussion

In the present study, we intended to evaluate the putative prognostic value of p53 expression and *RAS* mutations in cSCCs, since it remains a matter of controversy.

Our study indicates that *RAS* mutation seems to be more frequent in invasive rather than in situ cSCCs but studies with a larger number of in situ tumors will be pivotal to validate this hypothesis. When addressing invasive cSCCs, we report a similar *RAS* mutation rate (10.7%) in agreement with most of the previously-published studies (up to 13%) [26,39,40,41]. *RAS* mutations were more-frequently associated with an infiltrative than an expansive pattern of invasion, suggesting that these mutations might enhance tumor progression. A putative mechanism for a more infiltrative tumor front has been reported, suggesting that *RAS* mutations cooperate in modifying the epithelial-to-mesenchymal transition [42,43]. In our series, we observed a higher frequency of lymphovascular invasion in *RAS* mutated cSCCs, although not reaching significance.

As previously reported, no differences in p53 immunohistochemistry were observed in non-invasive and invasive cSCCs in the present study [16]. p53 overexpression detected (84%) is within the range of previous reports (15–92%) of invasive cSCCs [44,45,46,47]. No association was noted between p53 overexpression and clinicopathological features.

Our recurrence rate (13.0%) in invasive cSCCs is within the range of previously-reported studies (3.0–16.0%) [7,34,48,49,50]. In contrast with the reported studies, we observed a slightly higher lymph node metastasis rate (6.1% vs. 3.7–5.2%) [7,34,51,52]. The fact that 74.8% of invasive cSCCs were located on the face, including locations with higher metastatic risk, may in part explain the differences in the aforementioned reported rates.

*RAS* mutations were not associated with cSCC prognosis in our study. As previously reported by our group, older age and *TERTp* mutations turned out to be independent predictors of recurrence in invasive cSCCs [12]. In our study, ulceration was detected to be a predictor of recurrence in the univariate analysis. Although ulceration has been described as a risk factor for recurrence in melanoma [53], previous studies have failed to establish this premise in cSCCs [54,55]. Noteworthy, p53 overexpression was a predictor of recurrence in the univariate analysis, but previous studies failed to establish a prognostic significance [56]. When adjusted for other co-variables, the presence of ulceration and p53 overexpression failed to be independent predictors of recurrence in our study. Despite not being an independent predictor of recurrence, we must highlight that 16 out of 17 invasive cSCCs recurred and disclosed p53 overexpression. Further studies with a larger number of recurrent invasive cSCCs are warranted to ascertain the role of p53 overexpression as a putative biomarker of recurrence.

Invasion of the subcutaneous tissue and maximal tumor thickness are established risk factors for metastasis of cSCCs and were predictors of metastasis in our univariate analysis [11]. An association with metastasis and few or absent peritumoral lymphocytic infiltrate was detected in the univariate analysis and with a shorter time until adverse outcome. In other tumor models (e.g., melanoma), the absence or reduced number of lymphocytes (let alone the different subsets by immunohistochemistry) is an independent parameter associated with adverse prognosis [57,58,59]. Notwithstanding these results, when adjusted for other co-variables, invasion of the subcutaneous tissue, maximal tumor thickness, and few or absent peritumoral infiltrate failed to be independent prognostic predictors in our series of cSCCs. Importantly, p53 overexpression was detected in all metastatic cSCCs in our series. The limited number of metastases in our series might have hampered p53 overexpression prognostic significance in predicting metastasis of cSCCs.

The role of *RAS* mutations and p53 overexpression remains to be clarified in early cutaneous squamous cell carcinogenesis. *RAS* mutation rate did not differ when comparing in situ with invasive cSCCs and none of the three cases that recurred disclosed *RAS* mutation. p53 immunostaining was not significantly different in invasive and in situ cSCCs, but the three in situ cSCCs that recurred disclosed p53 overexpression. As in invasive cSCCs, the role of p53 overexpression as a marker of recurrence might be considered for in situ cSCCs, even though studies with a higher number of recurrent cases are mandatory.

The synergistic interaction between *RAS* mutation and p53 described in a mice model of cSCC was not confirmed in our series (see Supplementary Tables S5 and S6).

Several studies have suggested that cSCCs arising in lower extremities have distinctive features, including an increased frequency in women, as opposed to cSCCs in other localizations where there is a male preponderance [60,61]. Lower extremities are reported as the most common site for cSCCs in African Americans, suggesting a pathogenesis less reliant on chronic sun damage [62]. In our series, in situ cSCCs localized in the lower limbs were significantly more common in female than in male patients, but this difference was not detected in invasive cSCCs. Remarkably, we observed that in situ cSCCs located in the lower limbs had a significantly higher p53 h-score compared to other locations. To our knowledge, our results have not been previously described and support the hypothesis that in situ cSCCs might have distinct etiopathogenesis. Further studies and larger series will be important to cover the two major pitfalls we detected in this study, the lack of a considerable number of metastatic cSCCs with an extensive follow-up of patients, and the absence of *TP53* mutational genotyping to correlate with the p53 expression; as reported by Köbel and colleagues, it is not possible to predict the presence of *TP53* mutation based on its immunoexpression, since wild type and mutated cases can partially give rise to similar staining patterns [63].

## 5. Conclusions

Our results indicate that *RAS* mutations associate with features of local aggressiveness, but do not seem to be independent prognostic markers of outcome in patients affected by cSCC. All the metastatic cSCCs had p53 overexpression, and p53 overexpression was an independent predictor of recurrence in the univariate analysis. Larger studies with more recurrent and metastatic cSCCs are necessary to confirm the putative prognostic significance of p53 overexpression in patients’ risk stratification.

## Figures and Tables

**Table 1:** Clinicopathological features, frequency of *RAS* mutations, and p53 expression.

		All Tumors	In Situ cSCCs	Invasive cSCCs
***Clinical and molecular features***	**Number of cases**	162	31	131
	**Age at diagnosis (mean (±SD))**	77.6 ± 12.2	79.5 ± 7.4	77.1 ± 13.0
	Male	74.9 ± 12.2	78.8 ± 6.3	74.1 ± 12.9
	Female	81.6 ± 11.0	80.3 ± 8.6	82.0 ± 11.7
	**Gender (*n* (%))**			
	Male	97 (59.9)	16 (51.6)	81 (61.8)
	Female	65 (40.1)	15 (48.4)	50 (38.2)
	**Sun exposure (*n* (%))**			
	Chronic	110 (67.9)	11 (35.5)	99 (75.6)
	Intermittent	49 (30.2)	19 (61.3)	30 (22.9)
	Undetermined	3 (1.9)	1 (3.2)	2 (1.5)
	**Location (*n* (%))**			
	Face	108 (66.7)	10 (32.3)	98 (74.8)
	Trunk	9 (5.6)	5 (16.1)	4 (3.1)
	Upper limb	20 (12.3)	4 (12.9)	16 (12.2)
	Lower limb	22 (13.6)	11 (35.5)	11 (8.4)
	Not specified	3 (1.9)	1 (3.2)	2 (1.5)
	**Follow-up (months)**	41.6 ± 28.9	38.9 ± 21.5	42.2 ± 30.3
	**Progression-free survival (months)**	38.7 ± 29.2	37.6 ± 21.7	38.9 ± 30.7
	**Recurrence**			
	No	142 (87.7)	28 (90.3)	114 (87.0)
	Yes	20 (12.3)	3 (9.7)	17 (13.0)
	**Metastases**			
	No	154 (95.1)	31 (100)	123 (93.9)
	Yes	8 (4.9)	0	8 (6.1)
	***p53* immunohistochemistry**			
	Cells counted	4224,4 ± 2223.7	3041,1 ± 1066.0	4497,5 ± 2332.1
	Mean h-score	91.6 ± 5.9	109.6 ± 16.3	87.9 ± 6.2
	Wild type	29 (17.9)	8 (25.8)	21 (16.0)
	Overexpression (h-score 1+)	78 (48.1)	12 (38.7)	66 (50.4)
	Overexpression (h-score 2+)	45 (27.8)	8 (25.2)	37 (28.2)
	Overexpression (h-score 3+)	10 (6.2)	3 (9.7)	7 (5.3)
	***RAS* mutations**			
	Wild type	147 (90.7)	30 (96.8)	117 (89.3)
	Mutation	15 (9.3)	1 (3.2)	14 (10.7)
	***HRAS* mutations**			
	Wild type	149 (92.0)	30 (96.8)	119 (90.8)
	Mutation	13 (8.0)	1 (3.2)	12 (9.2)
	***KRAS* mutations**			
	Wild type	160 (98.8)	31 (100)	129 (98.5)
	Mutation	2 (1.2)	0 (0)	2 (1.5)
	***TERTp* mutations**			
	Wild type	98 (60.5)	21 (67.7)	77 (58.8)
	Mutation	48 (29.6)	6 (19.4)	42 (32.1)
***Maximum tumor thickness***	**Maximum tumor size**			
	<2 cm	75 (46.3)	12 (38.7)	63 (48.1)
	≥2 cm	39 (24.1)	9 (29.0)	30 (22.9)
	Not assessed	48 (29.6)	10 (32.3)	38 (29.0)
	**Superficial margins (mm)**	2.1 ± 2.8	1.7 ± 1.8	2.2 ± 2.9
	**Deep margins (mm)**	2.4 ±2.3	3.3 ± 1.7	2.2 ± 2.4
	**Ulceration**			
	No	53 (32.7)	11 (35.5)	42 (32.1)
	Yes	101 (62.3)	19 (61.3)	82 (62.6)
	Undetermined	8 (4.9)	1 (3.2)	7 (5.3)
	**Actinic Keratosis**			
	No	56 (34.6)	6 (19.4)	50 (38.2)
	Yes	96 (59.3)	25 (80.6)	71 (54.2)
	Undetermined	10 (6.6)		10 (7.6)
	**Invasion**			
	Non-invasive	31 (19.1)		
	Invasive	131 (80.9)		
	**Histologic type**			
	Acantholytic			10 (7.6)
	Spindle cell			1 (0.8)
	Verrucous			2 (1.5)
	Bowenoid			1 (0.8)
	Not otherwise specified (NOS)			117 (89.3)
	**Histological grade**			
	Well differentiated			46 (35.1)
	Moderately differentiated			68 (51.9)
	Poorly differentiated			13 (9.9)
	Not assessed			4 (3.1)
	**Pattern of invasion**			
	Expansive			70 (53.4)
	Infiltrative			57 (43.5)
	Not assessed			4 (3.1)
	**Level of invasion**			
	Papillary dermis			39 (29.8)
	Reticular dermis			60 (45.8)
	Subcutaneous tissue			26 (19.8)
	Not assessed			6 (4.6)
	**Maximum tumor thickness**			3.8 ± 3.0
	**Maximum tumor thickness**			
	< 6 mm			103 (78.6)
	≥ 6 mm			22 (16.8)
	Not assessed			6 (4.6)
	**Intratumoral infiltrate**			
	Moderate–intense			13 (9.9)
	Few–absent			118 (90.1)
	**Peritumoral infiltrate**			
	Moderate–intense			74 (56.5)
	Few–absent			57 (43.5)
	**Lymphovascular invasion**			
	Not present			126 (96.2)
	Present			5 (3.8)
	**Perineural invasion**			
	Not present			128 (97.7)
	Present			3 (2.3)

**Table 2:** Predictive factors for recurrence and lymph node metastasis.

	Recurrence				Metastasis			
	Univariate Analysis		Multivariate Analysis		Univariate Analysis		Multivariate Analysis	
	Odds Ratio (OR) (95% CI)	*p*	OR (95% CI)	*p*	OR (95% CI)	*p*	OR (95% CI)	*p*
**Mean age (years)**								
< 80	1	**0.008**	1	**0.019**	NA	NA		
≥ 80	16.00 (2.05–124.71)		12.17 (1.51–97.82)					
**Ulceration**								
No	1	**0.049**	1	0.261	1	0.196		
Yes	2.92 (1.00–8.51)		1.97 (0.60–6.41)		2.77 (0.59–13.01)			
**Level of invasion**								
**Dermis**	1	0.829			1	**0.028**	1	0.315
**Subcutaneous tissue**	0.86 (0.23–3.29)				5.82 (1.21–27.89)		2.68 (0.39–18.27)	
***RAS***								
Wild type	1	0.878			1	0.864		
Mutation	1.13 (0.23–5.57)				1.21 (0.14–10.62)			
*p53* overexpression *								
	1.01 (1.00–1.02)	**0.045**	1.01 (1.00–1.02)	0.145	1.01 (1.00–1.02)	0.281		
Superficial margins *								
	1.14 (1.00–1.31)	0.059			1.18 (1.02–1.37)	**0.026**	1.03 (0.85–1.25)	0.753
Max. tumor thickness *								
	1.09 (0.94–1.27)	0.238			1.25 (1.05–1.40)	**0.011**	1.17 (0.90–1.51)	0.247
**Peritumoral infiltrate**								
**Moderate–intense**	1	0.178			1	**0.032**	1	0.069
**Few–absent**	2.04 (0.72–5.74)				10.22 (1.22–85.65)		8.00 (0.85–75.30)	

*RAS* mutations and parameters with significant results in the univariate analysis in one of the adverse outcomes (recurrence or metastasis). All other clinicopathological features were not associated with outcome in the univariate analysis. NA, no metastasis occurred in patients <80 years of age. * p53 overexpression, superficial margins, and maximal tumor thickness were analyzed in the model as a continuous variable.

## Supplementary Materials

The following are available online at https://www.mdpi.com/2073-4425/11/7/751/s1, Table S1: Clinicopathological and molecular associations with RAS mutations in cSCC, Table S2: Clinicopathological and molecular associations with p53 overexpression, Table S3: Clinicopathological and molecular associations with p53 overexpression (mean h-score), Table S4: Clinicopathological and molecular associations with p53 overexpression (mean h-score) of cSCC with adverse features, Table S5: Features of cSCC with RAS mutated and with p53 overexpression, Table S6: Clinicopathological and molecular combinations between RAS status and p53 expression.
