# Supplementary material for: Prognostic Significance of RAS Mutations and P53 Expression in Cutaneous Squamous Cell Carcinomas
Source: Genes (Basel). 2020 Jul 6;11(7):751. doi: 10.3390/genes11070751 (PMC7397334; doi:10.3390/genes11070751)
Supplement: Supplementary file 1 [file genes-11-00751-s001.pdf]

Supplementary Materials:

Table S1: "Clinicopathological and molecular associations with *RAS* mutations in cSCC"

	**All tumors**				***in situ* cSCC**				**Invasive cSCC**			
Clinical pathological features	***Total***	***RAS WT***	***RAS Mut***	***p***	***Total***	***RAS WT***	***RAS Mut***	***p***	***Total***	***RAS WT***	***RAS Mut***	***p***
Number of cases	162	147 (90.7%)	15 (9.3%)		31	30 (96.8%)	1 (3.2%)		131	117 (89.3%)	14 (10.7%)	
**Age at diagnosis (mean (±SD))**	77.6 ± 12.2	78.0 ± 12.0	74.0 ± 13.9	0.231	79.5 ± 7.4	79.6 ± 7.5	77.0	0.736	77.1 ±13.0	77.5 ± 12.8	73.8 ± 14.4	0.310
Male	74.9 ± 12.2	75.1 ± 12.1	73.3 ± 13.2	0.621	78.8 ± 6.3	78.9 ± 6.6	77.0	0.779	74.1 ± 12.9	74.3 ± 12.9	72.9 ± 13.8	0.742
Female	81.6 ± 11.0	81.8 ± 10.7	77.0 ± 19.1	0.462	80.3 ± 8.6	80.3 ± 8.6	-	-	82.0 ± 11.7	82.3 ± 11.3	77.0 ± 19.1	0.449
Gender (n (%))												
Male	97 (59.9)	85 (57.8)	12 (80.0)	0.095	16 (51.6)	15 (50.0)	1 (100.0)	1.000	81 (61.8)	70 (59.8)	11 (78.6)	0.247
Female	65 (40.1)	62 (42.2)	3 (20.0)		15 (48.4)	15 (50.0)	0 (0.0)		50 (38.2)	47 (40.2)	3 (21.4)	
Sun exposure (n (%))												
Chronic	110 (67.9)	101 (68.7)	9 (60.0)	0.255	11 (35.5)	11 (36.7)	0	1.000	99 (75.6)	90 (76.9)	9 (64.3)	0.138
Intermittent	49 (30.2)	44 (29.9)	5 (33.3)		19 (61.3)	18 (60.0)	1 (100.0)		30 (22.9)	26 (22.2)	4 (28.6)	
Undetermined	3 (1.9)	2 (1.4)	1 (6.7)		1 (3.2)	1 (3.3)	0		2 (1.5)	1 (0.9)	1 (7.1)	
Location												
Face	108 (66.7)	99 (67.3)	9 (60.0)		10 (32.3)	10 (33.3)	0		98 (74.8)	89 (76.1)	9 (64.3)	
Trunk	9 (5.6)	8 (5.4)	1 (6.7)		5 (16.1)	4 (13.3)	1 (100.0)		4 (3.1)	4 (3.4)	0	
Upper limb	20 (12.3)	17 (11.6)	3 (20.0)	0.303	4 (12.9)	4 (13.3)	0	0.323	16 (12.2)	13 (11.1)	3 (21.4)	0.256
Lower limb	22 (13.6)	21 (14.3)	1 (6.7)		11 (35.5)	11 (36.7)	0		11 (8.4)	10 (8.5)	1 (7.1)	
Undetermined	3 (1.9)	2 (1.4)	1 (6.7)		1 (3.2)	1 (3.3)	0		2 (1.5)	1 (0.9)	1 (7.1)	
Location												
Extra-facial	54 (33.3)	48 (32.7)	6 (40.0)	0.775	21 (67.7)	20 (66.7)	1 (100.0)	1.000	33 (25.2)	28 (23.9)	5 (35.7)	0.341
Face	108 (66.7)	99 (67.3)	9 (60.0)		10 (32.3)	10 (33.3)	0		98 (74.8)	89 (76.1)	9 (64.3)	
Maximum tumor size (cm)	1.8 ± 1.4	1.8 ± 1.4	2.0 ± 1.3	0.665	1.6 ± 0.8	1.7±0.9	1.2	0.612	1.8 ± 1.4	1.8 ± 1.5	2.0 ± 1.3	0.632
Maximum tumor size												
< 2 cm	75 (46.3)	67 (65.0)	8 (72.7)	0.746	12 (38.7)	11 (55.0)	1 (100.0)	1.000	63 (48.1)	56 (67.5)	7 (70.0)	1.000
≥ 2 cm	39 (24.1)	36 (35.0)	3 (27.3)		9 (29.0)	9 (45.0)	0		30 (22.9)	27 (32.5)	3 (30.0)	
Not assessed	48 (29.6)				10 (32.3)				38 (29.0)			
Superficial margins (mm)	2.1 ± 2.8	2.0 ± 2.3	3.4 ± 5.6	0.363	1.7 ± 1.8	1.7 ± 1.8	2.0	0.872	2.2 ± 2.9	2.1 ± 2.4	3.5 ± 5.8	0.389
Deep margins (mm)	2.4±2.3	2.4 ± 2.4	1.9 ± 1.9	0.409	3.3 ± 1.7	3.2 ± 1.6	6.0	0.103	2.2 ± 2.4	2.3 ± 2.5	1.6 ± 1.6	0.320
Ulceration												
No	53 (32.7)	47 (33.8)	6 (40.0)	0.776	11 (35.5)	10 (34.5)	1 (100.0)	0.367	42 (32.1)	37 (33.6)	5 (35.7)	1.000
Yes	101 (62.3)	92 (66.2)	9 (60.0)		19 (61.3)	19 (65.5)	0		82 (62.6)	73 (66.4)	9 (64.3)	
Undetermined	8 (4.9)				1 (3.2)				7 (5.3)			

Actinic Keratosis												
No	96 (59.3)	88 (64.2)	8 (53.3)	0.574	6 (19.4)	6 (20.0)	0	1.000	50 (38.2)	43 (40.2)	7 (50.0)	0.568
Yes	56 (34.6)	49 (35.8)	7 (46.7)		25 (80.6)	24 (80.0)	1 (100.0)		71 (54.2)	64 (59.8)	7 (50.0)	
Undetermined	10 (6.2)								10 (7.6)			
Invasion												
Non-invasive	31 (19.1)	30 (20.4)	1 (6.7)	0.307								
Invasive	131 (80.9)	117 (79.6)	14 (93.3)									
Histological type												
Acantholytic									10 (7.6)	9 (7.7)	1 (7.1)	1.000
Spindle cell									1 (0.8)	1 (0.9)	0	
Verrucous									2 (1.5)	2 (1.7)	0	
Bowenoid									1 (0.8)	1 (0.9)	0	
NOS									117 (89.3)	104 (88.9)	13 (92.9)	
Histological grade												
Well-differentiated									46 (35.1)	42 (35.9)	4 (28.6)	0.511
Moderately differentiated									68 (51.9)	61 (52.1)	7 (50.0)	
Poorly differentiated									13 (9.9)	11 (9.4)	2 (14.3)	
Not assessed									4 (3.1)	3 (2.6)	1 (7.1)	
Histological grade												
Well-differentiated									46 (35.1)	42 (35.9)	4 (28.6)	0.382
Moderately-Poorly differentiated									81 (61.8)	72 (61.5)	9 (64.3)	
Not assessed									4 (3.1)	3 (2.6)	1 (7.1)	
Pattern of invasion												
Expansive									70 (53.4)	66 (58.4)	4 (28.6)	**0.046**
Infiltrative									57 (43.5)	47 (41.6)	10 (71.4)	
Not assessed									4 (3.1)			
**Level of invasion**												
Papillary dermis									39 (29.8)	35 (31.5)	4 (28.6)	0.350
Reticular dermis									60 (45.8)	55 (49.5)	5 (35.7)	
Subcutaneous tissue									26 (19.8)	21 (18.9)	5 (35.7)	
Not assessed									6 (4.6)			
Level of invasion												
Dermis									99 (75.6)	90 (81.1)	9 (64.3)	0.166
Subcutaneous tissue									26 (19.8)	21 (18.9)	5 (35.7)	
Not assessed									6 (4.6)			
Maximum tumor thickness									3.8±3.0	3.7 ± 2.8	4.8±4.5	0.192
Maximum tumor thickness												
< 6 mm									103 (78.6)	92 (82.9)	11 (78.6)	0.712
≥ 6 mm									22 (16.8)	19 (17.1)	3 (21.4)	
Not assessed									6 (4.6)			
**Intratumoral infiltrate**												
Moderate-intense									13 (9.9)	13 (11.1)	0	0.359

Few-absent									118 (90.1)	104 (88.9)	14 (100.0)	
**Peritumoral infiltrate**												
Moderate-intense									74 (56.5)	68 (58.1)	6 (42.9)	0.393
Few-absent									57 (43.5)	49 (41.9)	8 (57.1)	
**Lymphovascular invasion**												
Not present									126 (96.2)	114 (97.4)	12 (85.7)	0.088
Present									5 (3.8)	3 (2.6)	2 (14.3)	
**Perineural invasion**												
Not present									128 (97.7)	114 (97.4)	14 (100.0)	1.000
Present									3 (2.3)	3 (2.6)	0	
**Recurrence**												
No					28 (90.3)	27 (90.0)	1 (100.0)	1.000	114 (87.0)	102 (87.2)	12 (85.7)	1.000
Yes					3 (9.7)	3 (10.0)	0		17 (13.0)	15 (12.8)	2 (14.3)	
**Metastasis**												
No					31 (100)				123 (93.9)	110 (94.0)	13 (92.9)	1.000
Yes					0				8 (6.1)	7 (6.0)	1 (7.1)	
**Progression free survival (months)**	38.7 ± 29.2	38.2 ± 29.2	42.7 ± 29.8	0.577	37.6 ± 21.7	37.6 ± 22.1	36.0	0.943	38.9 ± 30.7	38.4 ± 30.8	43.1 ± 30.9	0.586
**Follow-up (months)**	41.6 ± 28.9	41.2 ± 29.1	45.3 ± 27.6	0.601	38.9 ± 21.5	39.0 ± 21.9	36.0	0.894	42.2 ± 30.3	41.8 ± 30.6	46.0 ± 28.5	0.624
TERTp mutations												
Wild type	98 (60.5)	91 (68.9)	7 (50.0)	0.229	21 (67.7)	21 (80.8)	0 (0)	0.222	77 (58.8)	70 (66.0)	7 (53.8)	0.540
Mutation	48 (29.6)	41 (31.1)	7 (50.0)		6 (19.4)	5 (19.2)	1 (100.0)		42 (32.1)	36 (34.0)	6 (46.2)	
Undetermined	16 (9.9)				4 (12.9)				12 (9.2)			
p53 expression												
Wild type	29 (17.9)	28 (19.0)	1 (6.7)	0.312	8 (25.8)	8 (26.7)	0	1.0	21 (16.0)	20 (17.1)	1 (7.1)	0.467
Overexpression	133 (82.1)	119 (81.0)	14 (93.3)		23 (74.2)	22 (73.3)	1 (100.0)		110 (84.0)	97 (82.9)	13 (92.9)	

Unpaired student's t-test was used to compare means in continuous variables; Chi-square and Fisher´s exact tests were applied to evaluate a possible association between categorical parameters.

Table S2: "Clinicopathological and molecular associations with p53 overexpression"

Clinical pathological features	**p53 overexpression**			**p53 overexpression**			**p53 overexpression**		
	**No**	**Yes**	***p***	**No**	**Yes**	***p***	**No**	**Yes**	***p***
Number of cases	29 (17.9)	133 (82.1)	-	8 (25.8)	23 (74.1)	-	21 (16.0)	110 (84.0)	-
**Age at diagnosis (mean (±SD))**									
<80	18 (62.1)	55 (41.4)	**0.042**	5 (62.5)	10 (43.5)	0.433	13 (61.9)	45 (40.9)	0.076
≥ 80	11 (37.9)	78 (58.6)		3 (37.5)	13 (56.8)		8 (38.1)	65 (59.1)	

Gender (n (%))									
Male	19 (65.5)	78 (58.6)	0.494	5 (62.5)	11 (47.8)	0.685	14 (66.7)	67 (60.9)	0.619
Female	10 (34.5)	55 (41.4)		3 (37.5)	12 (52.2)		7 (33.3)	43 (39.1)	
Sun exposure (n (%))									
Chronic	21 (72.4)	89 (66.9)	0.598	4 (50.0)	7 (30.4)	0.105	17 (81.0)	82 (74.5)	0.726
Intermittent	7 (24.1)	42 (31.6)		3 (37.5)	16 (69.6)		4 (19.0)	26 (23.6)	
Undetermined	1 (3.4)	2 (1.5)		1 (12.5)	0		0	2 (1.8)	
Location									
Face	20 (69.0)	88 (66.2)		4 (50.0	6 (26.1)		16 (76.6)	82 (74.5)	
Trunk	2 (6.9)	7 (5.3)		1 (12.5)	4 (17.4)		1 (4.8)	3 (2.7)	
Upper limb	2 (6.9)	18 (3.5)	0.830	1 (12.5)	3 (13.0)	0.229	1 (4.8)	15 (13.6)	0.592
Lower limb	4 (13.8)	18 (3.5)		1 (12.5)	10 (43.5)		3 (14.3)	8 (7.3)	
Undetermined	1 (3.4)	2 (1.5)		1 (12.5)	0		0	2 (1.8)	
Maximum tumor size (cm)	1.7 ± 1.0	1.8 ± 1.4	0.847	1.2 ± 0.6	1.8 ± 0.9	0.176	1.9 ± 1.0	1.8 ± 1.5	0.794
Maximum tumor size									
< 2 cm	13 (61.9)	62 (66.7)	0.678	4 (80.0)	8 (50.0)	0.338	9 (56.3)	54 (70.1)	0.280
≥ 2 cm	8 (38.1)	31 (33.3)		1 (20.0)	8 (50.0)		7 (43.8)	23 (29.9)	
Ulceration									
No	6 (21.4)	47 (37.3)	0.110	3 (37.5)	8 (36.4)	1.000	3 (15.0)	39 (37.5)	0.052
Yes	22 (78.6)	79 (62.7)		5 (62.5)	14 (63.6)		17 (85.0)	65 (62.5)	
Actinic Keratosis									
No	9 (34.6)	47 (37.3)	0.796	2 (25.0)	19 (82.6)	0.634	7 (38.9)	43 (41.7)	0.820
Yes	17 (65.4)	79 (62.7)		6 (75.0)	4 (17.4)		11 (61.1)	60 (58.3)	
Invasion									
Non-invasive	8 (27.6)	23 (17.3)	0.202				8 (27.6)	23 (17.3)	0.202
Invasive	21 (72.4)	110 (82.7)					21 (72.4)	110 (82.7)	
Histological type									
Acantholytic							1 (4.8)	9 (8.2)	
Spindle cell							0	1 (0.9)	0.193
Verrucous							0	2 (1.8)	
Bowenoid							1 (4.8)	0	
NOS							19 (90.5)	98 (89.1)	
Histological grade									
Well-differentiated							8 (38.1)	38 (34.5)	
Moderately differentiated							9 (42.9)	59 (53.6)	0.345
Poorly differentiated							4 (19.0)	9 (8.2)	
Not assessed							0	4 (3.6)	
Pattern of invasion									
Expansive							10 (50.0)	60 (56.1)	0.616

Infiltrative							10 (50.0)	47 (43.9)	
**Level of invasion**									
Papillary dermis							6 (33.3)	33 (30.8)	0.948
Reticular dermis							8 (44.4)	52 (48.6)	
Subcutaneous tissue							4 (22.2)	22 (20.6)	
Maximum tumor thickness									
< 6 mm							16 (84.2)	87 (82.1)	1.0
≥ 6 mm							3 (15.8)	19 (17.9)	
**Intratumoral infiltrate**									
Moderate-intense							1 (4.8)	12 (10.9)	0.692
Few-absent							20 (95.2)	98 (89.1)	
**Peritumoral infiltrate**									
Moderate-intense							15 (71.4)	59 (53.4)	0.132
Few-absent							6 (28.6)	51 (46.4)	
**Lymphovascular invasion**									
Not present							20 (95.2)	106 (90.4)	0.589
Present							1 (4.8)	4 (3.6)	
**Perineural invasion**									
Not present							20 (95.2)	108 (98.2)	0.411
Present							1 (4.8)	2 (1.8)	
**Recurrence**									
No				8 (100.0)	20 (87.0)	0.550	20 (95.2)	94 (85.5)	0.306
Yes				0	3 (13.0)		1 (4.8)	16 (14.5)	
**Metastasis**									
No				8 (100.0)	23 (100.0)	-	21 (100.0)	102 (92.7)	0.354
Yes				0	0		0	8 (7.3)	
TERTp mutations									
Wild type	18 (66.7)	80 (67.2)	0.955	5 (71.4)	16 (80.0)	0.633	13 (65.0)	64 (64.6)	0.976
Mutation	9 (33.3)	39 (32.8)		2 (28.6)	4 (20.0)		7 (35.0)	35 (35.4)	
RAS mutations									
Wild type	28 (96.6)	119 (89.5)	0.312	8 (100.0)	22 (95.7)	1.000	20 (95.2)	97 (88.2)	0.467
Mutation	1(3.4)	14 (10.5)		0	1 (4.3)		1(4.8)	13 (11.8)	

Chi-square and Fisher´s exact tests were applied to evaluate a possible association between parameters.

Table S3: "Clinicopathological and molecular associations with p53 overexpression (mean h-score)"

Clinical pathological features	p53 overexpression	*p*	p53 overexpression	*p*	p53 overexpression	*p*
Number of cases	133		23		110	
**Age at diagnosis (mean (±SD))**						
<80	85.9 ± 66.5	0.418	93.9 ± 63.4	0.413	84.2 ± 67.7	0.620
≥ 80	95.6 ± 68.6		121.5 ± 88.2		90.4 ± 63.5	

Gender (n (%))						
Male	88.4 ± 66.0	0.518	79.6 ± 54.4	0.073	89.9 ± 67.9	0.689
Female	96.2 ± 70.3		137.0 ± 88.1		84.8 ± 60.9	
Sun exposure (n (%))						
Chronic	88.5 ± 62.9	0.411	79.8 ± 54.7	0.235	89.3 ± 63.8	0.820
Intermittent	100.0 ± 77.6		122.5 ± 84.5		85.9 ± 71.1	
Location						
Face	88.0 ± 64.3		86.2 ± 56.9		88.1 ± 65.1	
Trunk	82.1 ± 77.4		57.5 ± 49.0	**0.024**	114.9 ± 107.6	
Upper limb	87.4 ± 62.2	0.343	51.4 ± 40.4		94.6 ± 64.3	0.794
Lower limb	121.4 ± 83.6		161.8 ± 78.0		70.9 ± 61.5	
Undetermined	55.5 ± 54.9		-		55.5 ± 54.9	
Maximum tumor size						
< 2 cm	104.4 ± 59.5	0.185	117.7 ± 63.3	0.988	102.5 ± 59.2	0.072
≥ 2 cm	85.7 ± 71.3		118.3 ± 77.9		74.4 ± 66.9	
Ulceration						
No	86.8 ± 68.6	0.691	104.6 ± 82.0	0.760	83.2 ± 66.1	0.793
Yes	91.7 ± 65.3		115.7 ± 80.4		86.5 ± 61.1	
Actinic Keratosis						
No	81.5 ± 64.1	0.202	90.4 ± 88.0	0.240	82.9 ± 66.6	0.553
Yes	97.1 ± 67.3		118.5 ± 82.7		90.4 ± 60.8	
Invasion						
Non-invasive	109.5 ± 78.0	0.163				
Invasive	87.9 ± 65.0					
Histological type						
Acantholytic					98.7 ± 76.5	
Spindle cell					36.8	
Verrucous					47.9 ± 27.5	0.659
Bowenoid					-	
NOS					88.2 ± 64.7	
Histological grade						
Well-differentiated					97.6 ± 72.1	
Moderately differentiated					83.7 ± 59.9	0.674
Poorly differentiated					84.0 ± 79.1	
Not assessed					66.3 ± 38.6	
Pattern of invasion						
Expansive					84.7 ± 63.6	0.757
Infiltrative					88.6 ± 64.3	
**Level of invasion**						
Papillary dermis					94.3 ± 65.9	
Reticular dermis					91.8 ± 60.7	0.243
Subcutaneous tissue					67.5 ± 62.3	

Maximum tumor thickness						
< 6 mm					90.6 ± 61.5	0.443
≥ 6 mm					78.4 ± 68.2	
**Intratumoral infiltrate**						
Moderate-intense					86.6 ± 56.8	0.941
Few-absent					88.0 ± 66.2	
**Peritumoral infiltrate**						
Moderate-intense					91.1 ± 64.2	0.580
Few-absent					84.2 ± 66.5	
**Lymphovascular invasion**						
Not present					87.2 ± 65.6	0.590
Present					105.2 ± 53.5	
**Perineural invasion**						
Not present					89.2 ± 64.9	0.114
Present					15.8 ± 12.2	
**Recurrence**						
No			112.2 ± 80.3	0.681	82.6 ± 61.5	**0.039**
Yes			91.7 ± 71.9		118.8 ± 78.0	
**Metastasis**						
No			109.6 ± 78.0	-	86.0 ± 62.8	0.280
Yes			-		111.9 ± 90.4	
TERTp mutations						
Wild type	93.1 ± 64.6	0.758	119.0 ± 84.2	0.543	86.7 ± 57.7	0.863
Mutation	89.1 ± 71.3		90.1 ± 72.8		89.0 ± 72.2	
RAS mutations						
Wild type	94.0 ± 69.2	0.242	108.8 ± 79.8	0.843	90.6 ± 66.5	0.229
Mutation	71.6 ± 50.5		125.2		67.4 ± 50.1	

Unpaired student's t-test was used to compare means in continuous variables; One-Way ANOVA was used to compare multiple co-variables; Chi-square and Fisher´s exact tests were applied to evaluate a possible association between categorical co-variables.

Table S4: "Clinicopathological and molecular associations with p53 overexpression (mean h-score) of cSCC with adverse features"

Case	Age / gender	Location	AO	PFS (months)	*RAS mutation*	H-score	Max (cm)	HT	HG	TT (mm)	Intratumoral infiltrate	Peritumoral infiltrate	LI/PI	FU (months)	Status at FU
1	85/F	Ear	R	22	WT	6.46	2	NOS	Mod	4	Few-abs	Mod-int	NI/NI	56	NED
2	78/M	Forehead	R	16	WT	89.39	1.8	NOS	Mod	1.5	Few-abs	Few-abs	NI/NI	22	DNR
3	80/M	Ear	R	35	WT	158.54	5	NOS	Mod	9	Mod-int	Mod-int	NI/NI	80	NED
4	81/M	Ear	R+Ms	11	WT	157.77	1.5	Acan	Mod	7	Mod-int	Few-abs	NI/NI	36	DOD
5	83/M	Forehead	R+Ms	9	WT	264.55	NA	NOS	Poor	NA	Few-abs	Few-abs	NI/NI	34	DNR
6	81/M	Ear	R	32	WT	173.10	0.9	NOS	Well	2	Few-abs	Mod-int	NI/NI	41	NED
7	80/F	Arm	R+Ms	28	WT	157.57	2.5	NOS	Well	2	Mod-int	Mod-int	NI/NI	96	NED
8	82/F	Hand	R+Ms	38	WT	148.49	1.2	NOS	Mod	5	Few-abs	Few-abs	P/NI	58	NED

9	75/M	Arm	R	39	WT	172.84	NA	NOS	Mod	3	Few-abs	Few-abs	NI/NI	56	NED
10	80/M	Temple	R	25	Mut	92.84	1.1	NOS	Mod	4	Few-abs	Few-abs	NI/NI	38	NED
11	86/M	Ear	Ms	3	WT	49.52	NA	NOS	Mod	2	Few-abs	Few-abs	NI/NI	12	DOD
12	87/M	Cheek	R	7	WT	145.07	1.5	NOS	Mod	4	Mod-int	Few-abs	NI/NI	24	DNR
13	87/F*	Cheek	R	12	WT	146.65	1	NOS	Mod	5	Few-abs	Mod-int	NI/NI	36	NED
14	87/F*	Forehead	R	12	WT	7.40	0.8	NOS	Mod	1.5	Few-abs	Mod-int	NI/NI	36	NED
15	88/F	Nose	R	16	WT	114.08	0.6	NOS	Mod	2	Few-abs	Few-abs	NI/NI	24	NED
16	82/F	Nose	R	20	WT	Wild type	0.9	NOS	Poor	3	Few-abs	Mod-int	NI/NI	62	NED
17	80/M	Ear	R	8	WT	204.35	3	NOS	Well	4	Few-abs	Few-abs	NI/NI	12	DNR
18	86/M	Ear	Ms	6	WT	82.66	NA	NOS	Mod	5	Few-abs	Few-abs	NI/NI	12	DNR
19	86/F	Nose	Ms	10	WT	10.00	2.5	NOS	Mod	11	Few-abs	Few-abs	NI/NI	24	NED
20	84/M	Scalp	R+Ms	3	Mut	6.05	NA	NOS	Mod	18	Few-abs	Few-abs	NI/NI	30	NED

Abbreviations: Acan – acantholytic, AO – adverse outcome, DNR – death not related, DOD – dead of disease, F – female, Few-abs – few-absent, FU – follow-up, HG – histologic grade, HT – Histological type, LI – lymphovascular invasion, M – male, Ms – lymph node metastasis, Max – maximum tumor size, Mod – moderately differentiated, Mod-int – moderate-intense, NA – not available/analyzed, NI – not identified, NED – no evidence of disease, NOS – not otherwise specified, PI – perineural invasion, P – present, PFS – progression-free survival, Poor – poorly-differentiated, R – recurrence, Sub – subcutaneous tissue, TT - tumor thickness, Well – well-differentiated, WT – wild type. *Tumor taken from the same patient on the same day.

Table S5: "Features of cSCC with *RAS* mutated and with p53 overexpression"

Case	Age / gender	Location	Sun exposure	Max (cm)	HT	HG	TT (mm)	Peritumoral infiltrate	Intratumoral infiltrate	LI/PI	Pattern invasion	*TERTp* mutation	AO
1	91/M	Forearm	Int	0.5	NOS	Mod	0.5	Few-abs	Few-abs	NI/NI	Inf	WT	-
2	92/M	Cheek	C	3.5	NOS	Poor	1.7	Mod-int	Few-abs	NI/NI	Inf	WT	-
3	80/M	Temple	C	1.1	NOS	Mod	4.0	Few-abs	Few-abs	NI/NI	Inf	Mut	R
4	77/M	Trunk	Int	1.2	NA	Mod	-	Mod-int	Few-abs	NI/NI	Inf	Mut	-
5	55/F	NA	NA	1.7	NOS	Mod	3.0	Few-abs	Few-abs	NI/NI	Inf	WT	-
6	77/M	Nose	C	0.8	NOS	Well	2.0	Few-abs	Few-abs	NI/NI	Exp	WT	-
7	73/M	Cheek	C	NA	NOS	Mod	4.0	Mod-int	Few-abs	NI/NI	Inf	Mut	-
8	47/M	Cheek	C	NA	Acan	Mod	5.0	Few-abs	Few-abs	P/NI	Inf	WT	-
9	59/M	Scalp	C	1.8	NOS	Poor	9.0	Mod-int	Few-abs	NI/NI	Inf	Mut	-
10	84/M	Scalp	C	NA	NOS	Mod	18.0	Few-abs	Few-abs	NI/NI	Exp	Mut	R+Ms
11	87/F	Arm	Int	4.0	NOS	Mod	7.0	Few-abs	Few-abs	P/NI	Exp	WT	-
12	89/F	Hand	Int	NA	NOS	IS	4.5	Few-abs	Few-abs	NI/NI	Inf	WT	-
13	71/M	Leg	Int	1.2	NOS	Well	3.5	Mod-int	Few-abs	NI/NI	Exp	WT	-
14	65/M	Chin	C	4.0	NOS	Well	0.5	Mod-int	Few-abs	NI/NI	Inf	Mut	-

Abbreviations: Acan – acantholytic, AO – adverse outcome, C – Chronic sun-exposure, Exp – expansive, F – female, Few-abs – few-absent, HG – histologic grade, HT – Histological type, Inf – infiltrative, Int – Intermittent sun-exposure, LI – lymphovascular invasion, M – male, Ms – lymph node metastasis, Max – maximum tumor size, Mod – moderately differentiated, Mod-int – moderate-intense, NA – not available/analyzed/applied, NI – not identified, NOS – not otherwise specified, PI – perineural invasion, P – present, Poor – poorly-differentiated, R – recurrence, TT - tumor thickness, Well – well-differentiated, WT – wild type.

Table S6: "Clinicopathological and molecular combinations between RAS status and p53 expression"

	All tumors					*in situ* cSCC				
Clinical pathological features	***RAS WT* / p53 *WT***	***RAS WT* / p53 overexpression**	***RAS mut* / p53 *WT***	***RAS mut* / p53 overexpression**	***p***	***RAS WT* / p53 *WT***	***RAS WT* / p53 overexpression**	***RAS mut* / p53 *WT***	***RAS mut* / p53 overexpression**	***p***
Number of cases	28 (17.3)	119 (73.5)	1 (0.6)	14 (8.6)	-	8 (25.8)	22 (71.0)	0	1 (100.0)	-
**Age at diagnosis (mean (±SD))**										
<80	17 (60.7)	47 (39.5)	1 (100.0)	8 (57.1)	0.071	5 (62.5)	9 (40.9)	-	1 (100.0)	0.313
≥ 80	11 (39.3)	72 (60.5)	0	6 (42.9)		3 (37.5)	13 (59.1)		0	
Gender (n (%))										
Male	18 (64.3)	67 (56.3)	1 (100.0)	11 (78.6)	0.299	5 (62.5)	10 (45.5)	-	1 (100.0)	0.554
Female	10 (35.7)	52 (43.7)	0	3 (21.4)		3 (37.5)	12 (54.5)		0	
Sun exposure (n (%))										
Chronic	20 (71.4)	81 (68.1)	1 (100.0)	8 (57.1)	0.357	4 (50.0)	7 (31.8)	-	0	0.191
Intermittent	7 (25.0)	37 (31.1)	0	5 (35.7)		3 (37.5)	15 (68.2)		1 (100.0)	
Undetermined	1 (3.6)	1 (0.8)	0	1 (7.1)		1 (12.5)	0		0	
Location										
Face	19 (67.9)	80 (67.2)	1 (100.0)	8 (57.1)	0.624	4 (50.0)	6 (27.3)	-	0	0.158
Trunk	2 (7.1)	6 (5.0)	0	1 (7.1)		1 (12.5)	3 (13.6)		1 (100.0)	
Upper limb	2 (7.1)	15 (12.6)	0	3 (21.4)		1 (12.5)	3 (13.6)		0	
Lower limb	4 (14.3)	17 (14.3)	0	1 (7.1)		1 (12.5)	10 (45.5)		0	
Undetermined	1 (3.6)	1 (0.8)	0	1 (7.1)		1 (12.5)	0		0	
Location*										
Extra-facial	9 (32.1)	39 (32.8)	0	6 (42.9)	0.834	4 (50.0)	16 (72.7)	-	0	0.583
Face	19 (67.9)	80 (67.2)	1 (100.0)	8 (57.1)		4 (50.0)	6 (27.3)		1 (100.0)	
Maximum tumor size (cm)	1.7 ± 1.0	1.8 ± 1.4	1.8	2.0 ± 1.3	0.974	1.2 ± 0.6	1.8 ± 0.9	-	1.2	0.320
Maximum tumor size										
< 2 cm	12 (60.0)	55 (66.3)	1 (100.0)	7 (70.0)	0.868	4 (80.0)	7 (46.7)	-	1 (100.0)	0.441
>= 2 cm	8 (40.0)	28 (33.7)	0	3 (30.0)		1 (20.0)	8 (53.3)		0	
Ulceration										
No	6 (22.2)	41 (36.6)	0	6 (42.9)	0.388	3 (37.5)	7 (33.3)	-	1 (100.0)	0.492
Yes	21 (77.8)	71 (63.4)	1 (100.0)	8 (57.1)		5 (62.5)	14 (66.7)		0	
Actinic Keratosis										
No	9 (36.0)	40 (35.7)	0	7 (50.0)	0.722	2 (25.0)	4 (18.2)	-	0	0.714
Yes	16 (64.0)	72 (64.3)	1 (100.0)	7 (50.0)		6 (75.0)	18 (81.8)		1 (100.0)	
Invasion										
Non-invasive	8 (28.6)	22 (18.5)	0	1 (7.1)	0.406					
Invasive	20 (71.4)	97 (81.5)	1 (100.0)	13 (92.7)						
Histological type										
Acantholytic										
Spindle cell										
Verrucous										
Bowenoid										
NOS										
Histological grade										
Well-differentiated										
Moderately differentiated										
Poorly differentiated										
Not assessed										
Pattern of invasion										
Expansive										
Infiltrative										
**Level of invasion**										
Papillary dermis										
Reticular dermis										
Subcutaneous tissue										
Level of invasion										
Dermis										
Subcutaneous tissue										
Maximum tumor thickness										
< 6 mm										
≥ 6 mm										
**Intratumoral infiltrate**										
Moderate-intense										
Few-absent										

**Peritumoral infiltrate**										
Moderate-intense										
Few-absent										
**Lymphovascular invasion**										
Not present										
Present										
**Perineural invasion**										
Not present										
Present										
**Recurrence**										
No						8 (100.0)	19 (86.4)	-	1 (100.0)	0.589
Yes						0	3 (13.6)		0	
**Metastasis**										
No						8 (100.0)	22 (100.0)	-	1 (100.0)	-
Yes						0	0		0	
TERTp mutations										
Wild type	18 (69.2)	73 (68.9)	0	7 (53.8)	0.373	5 (71.4)	16 (84.2)	-	0	0.152
Mutation	8 (30.8)	33 (31.1)	1 (100.0)	6 (46.2)		2 (28.6)	3 (15.8)		1 (100.0)	

One-way ANOVA was used to compare means in continuous variables; Chi-square and Fisher´s exact tests were applied to evaluate a possible association between categorical parameters.
